# Quality of life should be the primary outcome for disease modifying therapy trials in MS—No

**DOI:** 10.1177/13524585231182708

**Published:** 2023-07-25

**Authors:** EMM Strijbis, MW Koch, BA de Jong

**Affiliations:** Department of Neurology, MS Center Amsterdam, Amsterdam UMC, Amsterdam, the Netherlands; Department of Community Health Sciences, University of Calgary, Calgary, AB, Canada; Department of Neurology, MS Center Amsterdam, Amsterdam UMC, Amsterdam, the Netherlands

There is an ongoing call to incorporate patients’ views on their disease as outcomes in studying interventions in multiple sclerosis (MS).^1^ The expanding possibilities for intervening in the course and symptom burden of MS have led to a significant change in our treatment goals. Instead of hoping to control disease activity to a certain extent, we are increasingly aiming for the prevention of any disability through early and aggressive treatment. With the increasing complexity in treatment choices also comes the question how our treatment strategies affect health-related quality of life (HRQoL) of people with MS (pwMS).

Patient-based measures have the potential to capture various aspects of patients’ concerns about treatment burden, symptom alleviation, and disease control. They all fall under the overarching concept of HRQoL.^2^ While it is increasingly acknowledged that it is essential to complement standard clinical outcome measures with patients’ experiences and perspectives on their disease, HRQoL is a challenging construct to measure, especially in clinical trials. First, there are varying definitions of HRQoL, and as a consequence of this, there are still a wide variety of patient-reported outcome measures (PROMs) in use to measure this construct. Second, HRQoL measures were designed as cross-sectional measures; their use as longitudinal outcomes in clinical trials is not widely researched and made difficult by the clinimetric properties of the majority of these instruments.

Many different generic or disease-specific HRQoL instruments are applied in MS-related randomized controlled trials and registries. There are disease-specific instruments such as the Multiple Sclerosis Quality of Life-54 instrument and generic instruments that are used across different conditions. They include the commonly used Medical Outcomes Study short form-36 (SF-36) or the EuroQOL 5-Dimensions questionnaire. Additional measures of performance, specific symptoms, or proxies for disability are frequently used under the umbrella of HRQoL. Examples are the Multiple Sclerosis Impact Scale-29 (MSIS-29) or symptom-specific measures such as the MS Walking Scale or the Hospital Anxiety and Depression Scale. While all these instruments are part of the HRQoL construct, each one of them measures a different aspect of HRQoL. For the use of such measures in clinical trials, a precise a priori definition of the concepts patients and clinicians want to assess is essential to select the most suitable instrument to measure the domains that the interventions are addressing. A standard set of PROMs might be useful for the translation of results across trials.

In addition to the issue what exactly we want to measure, there is also the issue of how well we are able to measure it. The World Health Organization’s definition of HRQoL “*an individual’s perception of their position in life in the context of the culture and value systems in which they live and in relation to their goals, expectations, standards and concerns*” implies that QoL is determined by many more factors in addition to the disease process alone, including cultural differences and personal changes throughout life. Much of the research on the psychometric properties of most popular PROMs in MS comes from older cross-sectional studies mostly performed in Western countries, especially the UK and US. There are important issues in translating and adapting instruments across cultures that can potentially lead to cross-cultural differences in instruments.^3^ Cross-cultural differences in the HRQoL concept have led to different results in HRQoL studies.^4^ This has important implications for the generalizability of trial results to patients who do not fit the original profile of the study population.

Related to this, PROMs are subjective measures that are affected by patient’s internal factors such as mood and expectations and external factors such as treatment context, interaction with health care providers, and socioeconomic status. These issues are of particular concern in open-label trials, but these factors can of course also introduce “noise” in the longitudinal measurements in blinded settings. In clinical trials, these effects may have an even larger impact than that in real-life settings since internal and external factors are different in a trial setting, potentially introducing natural regression to the mean which might be interpreted as a treatment effect.^5^ Another issue of this subjectivity is response shift. The response shift phenomenon is described in at least 20% of patients, and it refers to the process where the appraisal of an individual’s internal standard of wellbeing changes over time.^6^ This phenomenon may influence HRQoL scores over time in pwMS, including in those participating in clinical trials. Another issue is that minimal important differences are dependent of disease characteristics and PROM scores at baseline. In addition, the translation from group-level changes to individual-level changes is still difficult and needs more research.^7^

A recent study in a large phase 3 clinical data set in SPMS highlights these issues.^8^ In this study, SF-36 and MSIS-29 scores changed little over 2 years of follow-up. While there was a steady worsening in physical disability measures (as might be expected in a population of people with SPMS), significant worsening on the SF-36 and MSIS-29 occurred about as often as improvement. This disconnect between the worsening physical disability and these two widely used HRQoL measures suggest that the underlying disease process of SPMS is not well reflected in these HRQoL measures. Consequently, a clinical trial using these measures as primary outcomes would be unable to detect whether the trialled treatment has an effect on the disease process of secondary progressive MS (SPMS).

Given these concerns, we cannot recommend the use of currently available HRQoL measures as primary outcomes in clinical trials of interventions in MS. We will need to design better HRQoL outcome measurers that reflect the disease course of MS combined with a reliable measure of HRQoL in a way that results can also be generalized to other populations. Until such outcome measures are available, HRQoL measures remain useful as ancillary outcome measures in clinical trials in MS.
